# The current trend of exosome in epithelial ovarian cancer studies: A bibliometric review

**DOI:** 10.3389/fphar.2023.1082066

**Published:** 2023-03-09

**Authors:** Neda Baghban, Mujib Ullah, Iraj Nabipour

**Affiliations:** ^1^ The Persian Gulf Marine Biotechnology Research Center, The Persian Gulf Biomedical Sciences Research Institute, Bushehr University of Medical Sciences, Bushehr, Iran; ^2^ Institute for Immunity and Transplantation, Stem Cell Biology and Regenerative Medicine, School of Medicine, Stanford University, Palo Alto, CA, United States; ^3^ Department of Cancer Immunology, Genentech Inc., South SanFrancisco, CA, United States; ^4^ Molecular Medicine Department of Medicine, Stanford University, Palo Alto, CA, United States

**Keywords:** MicroRNAs, female, cancer, ovarian, extracellular vesicle

## Abstract

**Background:** Epithelial ovarian cancer (EOC) is the most common type of ovarian cancer. About 90% of ovary tumors are epithelial. The current treatment for EOC involves surgical debulking of the tumors followed by a combination of chemotherapy. While most patients achieve complete remission, many EOCs will recur and develop chemoresistance. The cancer cells can adapt to several stress stimuli, becoming resistant. Therefore, new ways to fight resistant cells during the disease are being studied. Recently, exosomes, which reflect cell behavior in normal and pathological conditions such as epithelial ovarian cancer, are of academic interest as new biomarkers for diagnosis and therapy. Consequently, the current study aimed to investigate the research output of exosomes in EOC.

**Method:** A bibliometric method was used for analyzing publications on exosome and epithelial ovarian cancer from the beginning to 15 October 2022 by searching keywords in Scopus, PubMed and Google scholar. Annual scientific publications, authors, citations, journals, co-authorships, and keywords co-occurrence were analyzed and plotted using Microsoft Office Excel and VOS viewer. 39 original journal articles and 3 reviews have been published since 2015 up to 15 October 2022.

**Results:** The findings showed that China is the top country in research output, international collaborations, organization, author, and sponsorship. The top journals were the Journal of Ovarian Research, Oncotarget, and Tumor Biology, all in the United States. The top institution was Shanghai Jiao Tong University in China. The top author was Xipeng Wang. Co-occurrence analysis showed that academics’ interest is toward:1) 1) Exosomes as prognostic biomarkers of EOC as well as their role in the proliferation and migration of cells. 2) The role of exosomes in metastasis through different mechanisms; 3) The role of exosomes in epithelial-mesenchymal transition of ovarian cancer cells; 4) The diagnostic role of EVs in EOC; and 5) Conferring chemoresistance in EOC through the exosomal transfer of miRNAs.

**Conclusion:** Research on the exosome and EOC has an increasing trend, and China is much more involved than other countries in research, financial support, and international cooperation. These findings could aid researcher in understanding novel ideas and subjects interested by sponsors in this field.

## 1 Introduction

Despite decades of efforts for improving the effectiveness of treatment approaches, epithelial ovarian cancer (EOC) is still the most lethal of all gynecological malignancies (Obermair et al., 2021). From a clinical point of view, the standard approach of EOC management is still debulking surgery and chemotherapy (Gadducci et al., 2022; Lodewijk et al., 2022). However, innovative surgical and medical developments were associated with only marginal survival improvements. The negative prognosis of patients suffering from OCE is normal because of late-stage diagnosis and the chemoresistance development during the illness period (Ma et al., 2014; Akter et al., 2022; Wanyama et al., 2022). Overdiagnosed, EOC remains an asymptomatic disease until the development of diffuse peritoneal carcinomatosis, including abdominal disease (van Baal et al., 2018). In addition, the transvaginal ultrasound combined with tumor markers carbohydrate antigen 125 (CA-125) serum level dosage as a promising screening method attracted attention, but yet had limitations in identifying EOC as an early-stage disease (Fishman et al., 2005; Olivier et al., 2006). Accordingly, 70% of patients diagnosed with EOC are frequently at the end stage of the disease with a 5-year survival rate of less than 40% (Meng et al., 2016a), so the necessity of developing non-invasive tools with highly sensitive for ensuring an early-stage diagnosis is one of the main challenges in biomarker investigate. Drug resistance development is another significant point in EOC investigations (Liu et al., 2018; Bukowski et al., 2020). In this regard, new advanced methods have been presented to date, precise approaches for detecting and treating different cancers (Bartosh et al., 2016; Baghban et al., 2022; Miri et al., 2022). One of the most newly known biomarkers of diseases is extracellular nano-vesicles, called exosomes (Hubert et al., 2015; Nawaz et al., 2016; Yousafzai et al., 2018; Ghafourian et al., 2022). Exosomes are produced by cells and carry several genetic materials and proteins playing key roles in the signaling and crosstalk of cells (Mashouri et al., 2019; Afshar et al., 2021; Bayat et al., 2021; Zhankina et al., 2021; Akbar et al., 2022). It has been reported that exosomes and their cargoes are an appropriate tool for maintaining the homeostasis of cancer tissues as they are able to mediate intercellular communication (Salido-Guadarrama et al., 2014; Caruso Bavisotto et al., 2019). Moreover, researchers propose that a panel of exosome-derived circulating miRNAs may aid to come for diagnosing early-onset of cancer and monitoring disease over cancer therapy (Wang and Chen, 2014). In this regard, increasing attention has been concentrated on the function of exosomes and their molecular cargo in EOC (Li and Wang, 2017).

In light of the above-mentioned, the present bibliometrics study focuses on analyzing and examining the scientific productivity developed specifically on exosomes in EOC up to 15 October 2022 to observe its evolution. In this context, the scientific productivity by year, countries, subject areas, organizations, sponsors, authors, citations, keyword co-occurrence, and co-authorships mapping related to exosomes and EOC were extracted from the titles and keywords of the included documents and analyzed. According to the best of our knowledge, there is no similar study on the subject of the exosome in EOC. The findings and statistics attained are of great value for academics working on this topic or beginning their initial steps.

## 2 Methods

**FIGURE 1:**
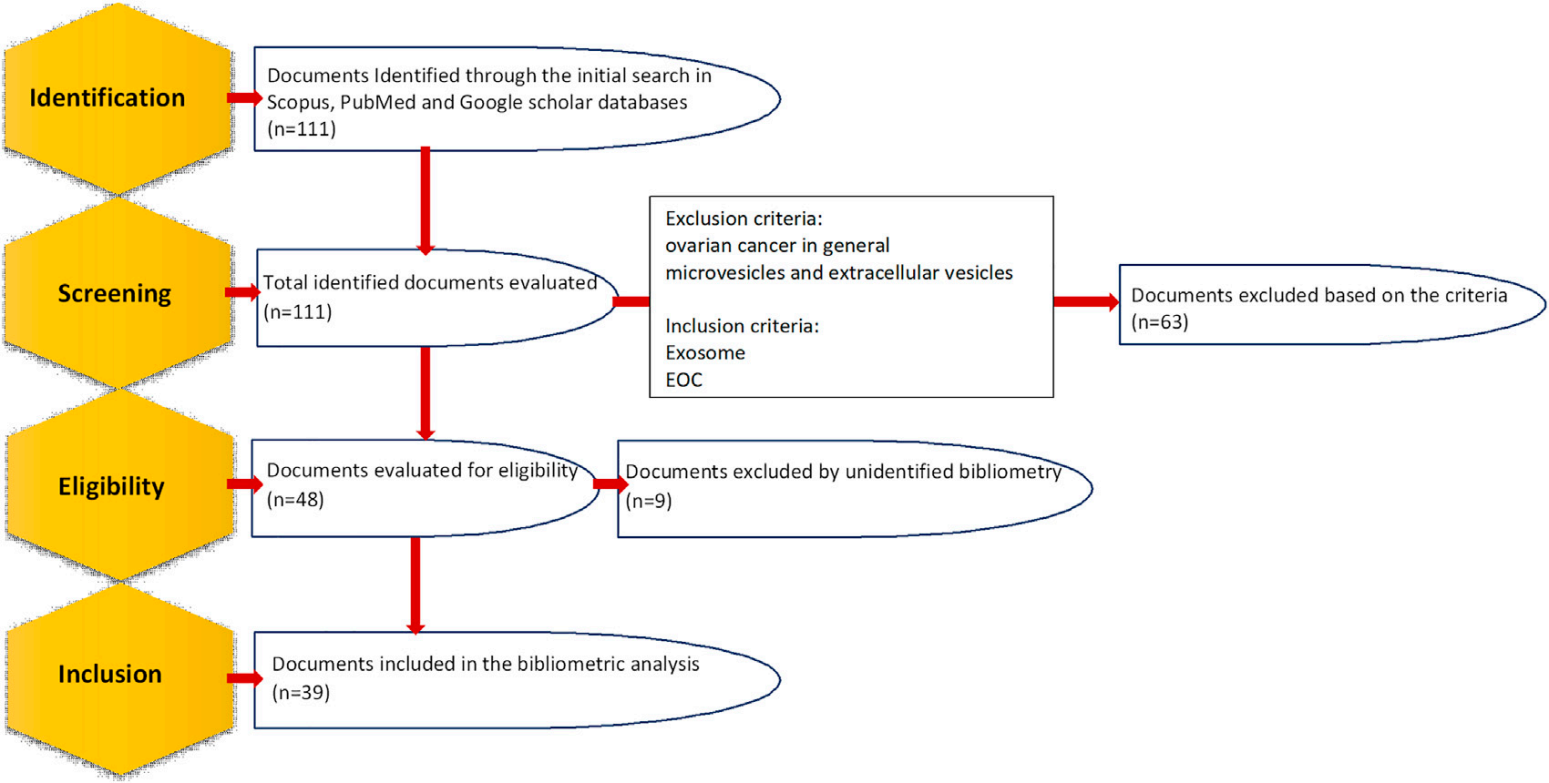
Steps of identifying and selecting documents according to preferred reporting items for systematic reviews and meta-analyses (PRISMA) guidelines.

The current research focuses on bibliometric analysis, which applies methods and software that allow identification, documentation and combination of diverse properties of the knowledge area (Donthu et al., 2021). Scientific mapping is performed according to the obtained data and different networks are resulted that define their relations between diverse factors. The proper expression of these networks will determine the upcoming lines of strengthening the scientific production of this field. Documents are tracked and selected according to the Preferred Reporting Items for Systematic Reviews and Meta-Analyses (PRISMA) guidelines (Figure 1) (Moher et al., 2009). The data for this study, dating from the beginning to 2022 (15 October 2022), will be extracted using the PoP software (version 8) from Scopus, PubMed and Google scholar using the following search terms in title and keywords: “epithelial ovarian cancer” and “exosomes” or “exosome” or “exosomal” or “extracellular vesicles” or “extracellular vesicle”. Then, those articles investigating ovarian cancer in general (which also includes epithelial ones) or microvesicles and extracellular vesicles (which also contain exosomes) were excluded as this study aims to investigate the studies focusing on only EOC and only exosomes. All the information including metadata on citation information and abstract, among other information will be exported in CSV format to the Microsoft Office Excel software for data analysis after merging the obtained files. Additionally, the VOSviewer software bibliometric analysis software, which enabled to build diverse illustrations of scientific mapping, will be employed to generate the collaboration and word co-occurrence networks.

## 3 Results

### 3.1 Scientific productivity by years and subject areas

**FIGURE 2:**
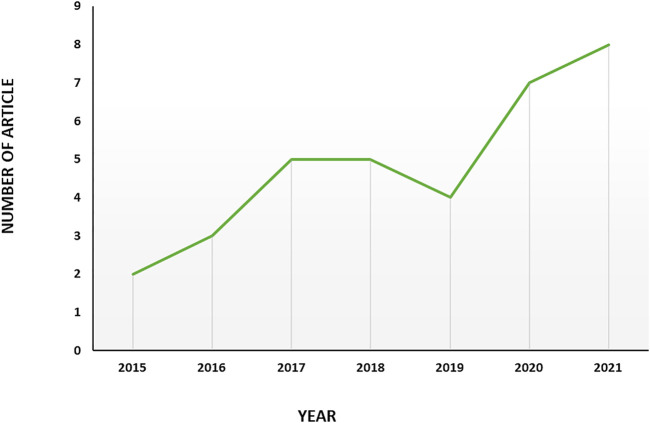
Trend of productivity by years in terms of number of publications in the field of exosome and EOC.

The primary research yielded 111 documents in Scopus, PubMed and Google scholar. Only articles published in English were included. After the exclusion of duplicate, irrelevant journal articles and non-journal articles, 39 articles on exosome and EOC were selected for bibliometric analysis; 36 of which were original articles (Zhang et al., 2016a; Meng et al., 2016b; Zhang et al., 2016b; Labani-Motlagh et al., 2016; Ying et al., 2016; Chen et al., 2017; Hu et al., 2017; Li et al., 2017; Wu et al., 2017; Chen et al., 2018; Qiu et al., 2018; Zhang et al., 2018; Zhou et al., 2018; Zhang et al., 2019a; Keserű et al., 2019; Tang et al., 2019; Zhu et al., 2019; Cheng et al., 2020; Li et al., 2020; Lu et al., 2020; Luo and Gui, 2020; Maeda et al., 2020; Masoumi-Dehghi et al., 2020; Alharbi et al., 2021; Xiong et al., 2021a; Cai et al., 2021; Gao et al., 2021; Li et al., 2021; Liu et al., 2021; Ma et al., 2021; Zhu et al., 2022a; Chen et al., 2022; Jeon et al., 2022; Lai et al., 2022; Yang et al., 2022), and the rest of them were review articles (Li and Wang, 2017; Lucidi et al., 2020; Shiao et al., 2021). The first English article is “Characterization of exosomes derived from ovarian cancer cells and normal ovarian epithelial cells by nanoparticle tracking analysis” in 2015 (Zhang et al., 2016a). The scientific production evolution has been illustrated in Figure 2. 2021 is the year with the greatest scientific production with 8 articles and a 4-fold increase in publication compared to the first year, 2015. In the case of 2022, as the year is not completed it is soon to judge about.

**FIGURE 3:**
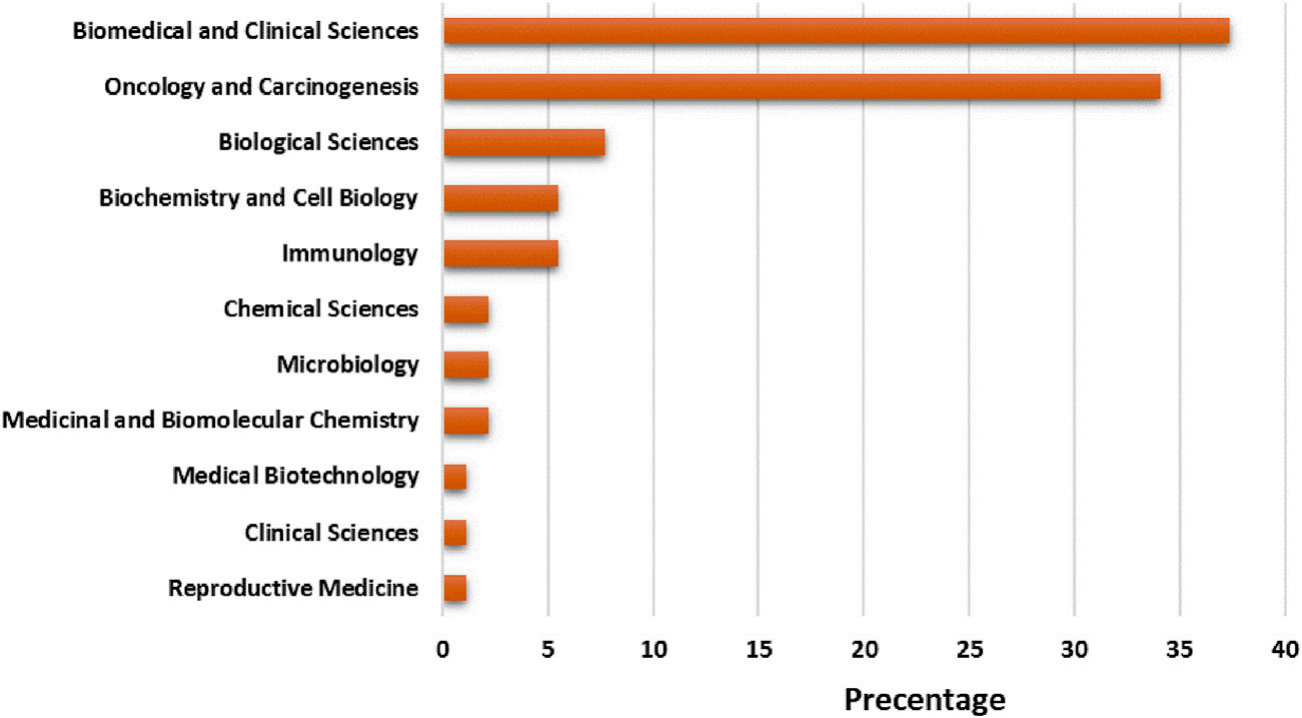
Trend of productivity by subject areas in terms of number of publications in the field of exosome and EOC.

Selected articles on exosome and OEC has been published in the context of 11 areas based on area categories of the Dimension database. “Biomedical and clinical sciences” has the maximum publication share (37.36%), followed by “oncology and carcinogenesis” (34.06%), “biological sciences” (6.50%), “biochemistry and cell biology” (4.58%), “multidisciplinary” (3.30%) “multidisciplinary” (3.30%) “multidisciplinary” (7.69%), “biochemistry and cell biology” and “immunology” (5.49%), “chemical sciences”, “microbiology” and “medicinal and biomolecular chemistry” (2.20%) and “medical biotechnology”, “clinical sciences” and “reproductive medicine” (1.10%) (Figure 3).

### 3.2 Countries

**FIGURE 4:**
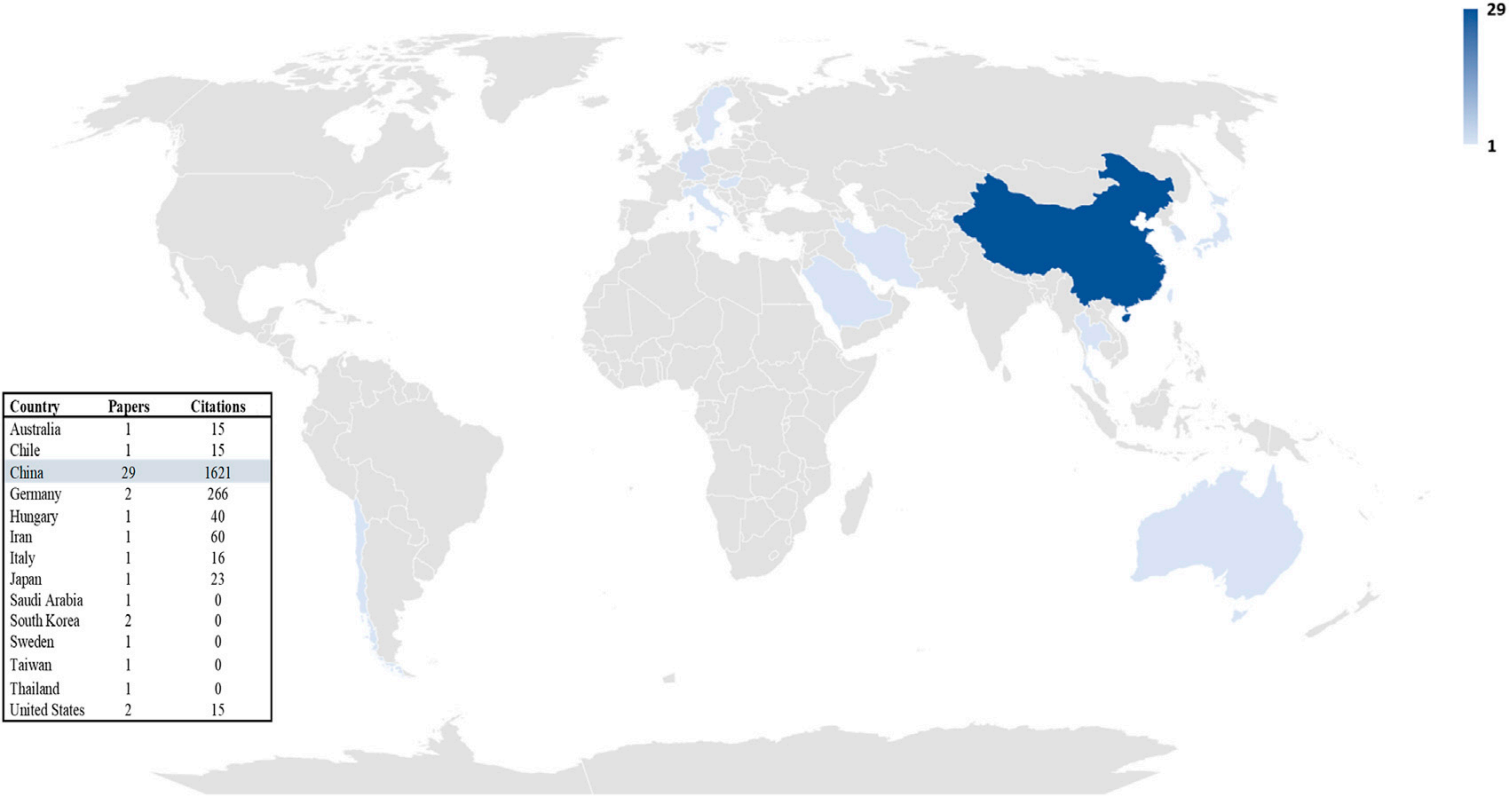
Trend of productivity by countries in terms of number of publications in the field of exosome and EOC.

A total of 14 countries including China, Germany, Sweden, Hungary, Japan, the United States, Italy, Iran, Thailand, South Korea, Taiwan, Australia, Saudi Arabia, and Chile had published at least one article related to the exosome and EOC from 2015 to 2022. As observed in Figure 4, China with 29 is the top country.

**FIGURE 5:**
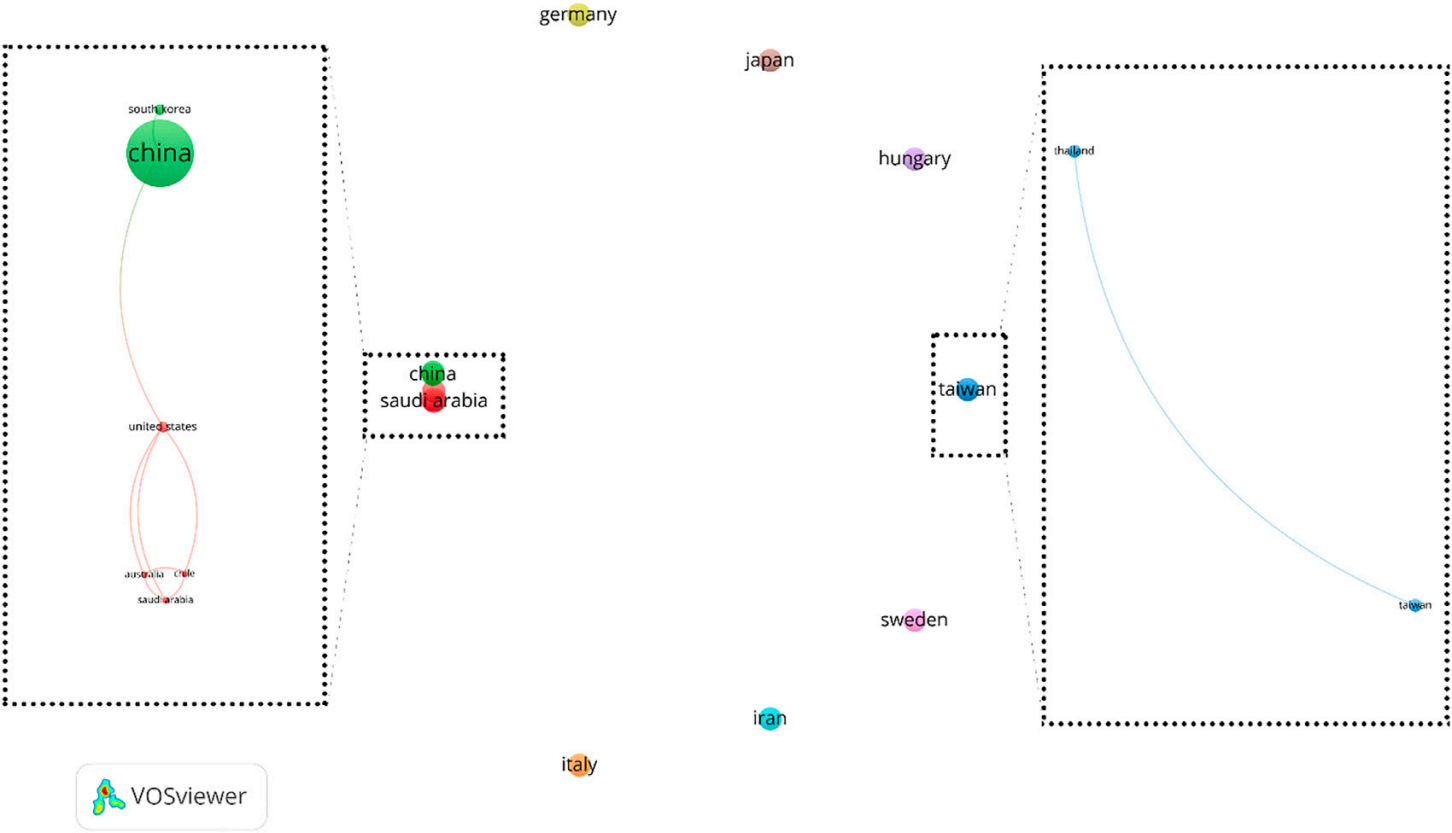
Country co-authorship networks (criteria: countries with at least 1 article in the field of exosome and OEC).

In order to analyze country co-authorship or international collaboration, all 14 countries were included in the analysis. This analysis resulted in 9 clusters that 3 of which contains only two or more country. These three clusters contain 8 countries and 2 out of these 3 clusters are linked together. These results have been illustrated in Figure 5. It shows that authors in eight countries have international collaboration. Among these countries, China has the best international collaboration.

### 3.3 Organizations

A total of 63 organizations including 25 hospitals and 38 universities and colleges published at least one journal article from 2015 up to 15 October 2022. Among these organizations, the Shanghai Jiao Tong University in China with 6 articles is ranked as the first producer university and the Tongji University in China with 5 articles is ranked as the second one. Table 1 shows the ranking of organizations with more than one journal article in this field between 2015 to 15 October 2022.

**TABLE 1:** Trend of productivity by institutions.

Organization	Country	Papers	Citations
Shanghai jiao tong university	China	6	599
Tongji university	China	5	671
Renji hospital	China	4	436
Xinhua hospital	China	4	434
University medical center hamburg-eppendorf	Germany	2	266
Affiliated hospital of jiangsu university	China	2	186
Jiangsu university	China	2	186
Fudan university	China	2	118
Huazhong university of science and technology	China	2	101
Tongji hospital	China	2	101
Chinese academy of medical sciences and peking union medical college	China	2	79
Peking union medical college hospital	China	2	79
Fudan university shanghai cancer center	China	2	78

**FIGURE 6:**
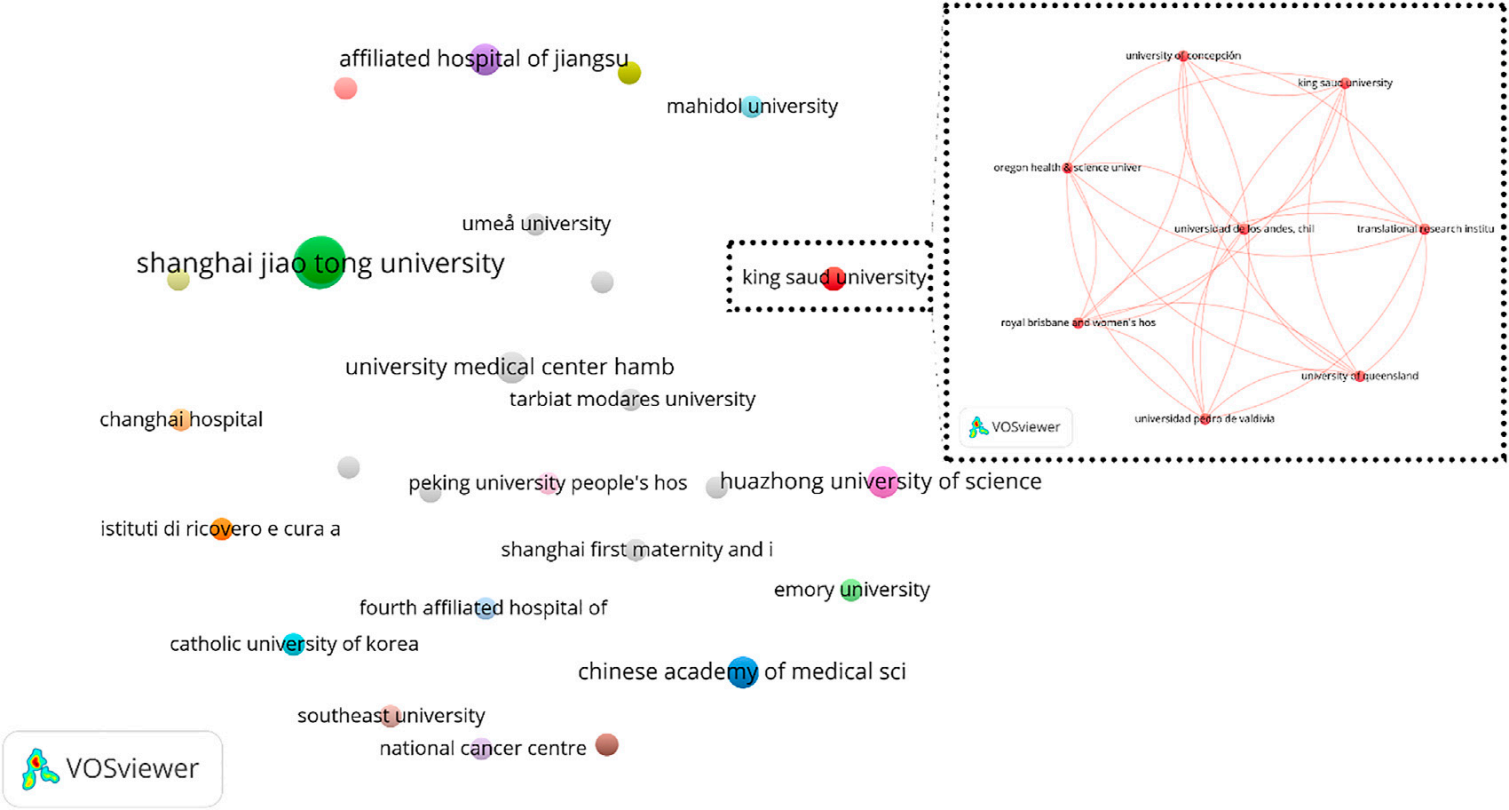
Organization co-authorship network (criteria: organizations with at least 1 article in the field of exosome and OEC).

To analyze the organizations’ collaboration, the organizations’ co-authorship network was obtained using VOSviewer software (Figure 6). All 63 identified organizations were included in the primary analysis and it resulted in 26 clusters that 9 of them has no collaboration with other organizations. The largest cluster contains 8 organizations including, which has been shown in detail in Figure 6.

### 3.4 Authors

245 authors are identified in 39 articles implying a productivity index of 0.16 articles per author. Authors with more than 2 articles in the field of exosome and EOC have been presented in Table 2. Xipeng Wang (Ying et al., 2016; Chen et al., 2017; Li and Wang, 2017; Wu et al., 2017; Chen et al., 2018; Zhou et al., 2018; Li et al., 2020), Xinjing Wang (Ying et al., 2016; Chen et al., 2017; Wu et al., 2017; Chen et al., 2018; Zhou et al., 2018; Li et al., 2020), and Qinyi Zhu (Ying et al., 2016; Chen et al., 2017; Wu et al., 2017; Zhou et al., 2018; Li et al., 2020) are the most productive author with 7, 6, and 5 articles in the field of exosome and EOC. Most citations are also related to the same authors with values of 887, 811, and 651, respectively. It is worth notable that these authors are in the same group.

**TABLE 2:** Most productive authors in terms of number of publications in the field of exosome and EOC.

Author	Papers	Citations
xipeng wang	7	887
xinjing wang	6	811
qinyi zhu	5	651
xin chen	4	601
xiaoduan li	4	446
xiaoli wu	4	627
yingying lin	3	370
xiang ying	3	441
wei zhang	3	143

Regarding the author collaboration trend in producing journal articles, there is no single authorship document and all articles display a contribution of two or more authors that authors in 25 of which have different affiliations. The participation of several authors with diverse affiliations in one manuscript displays thematic maturity (Berelson, 1952; López López, 1996).

**FIGURE 7:**
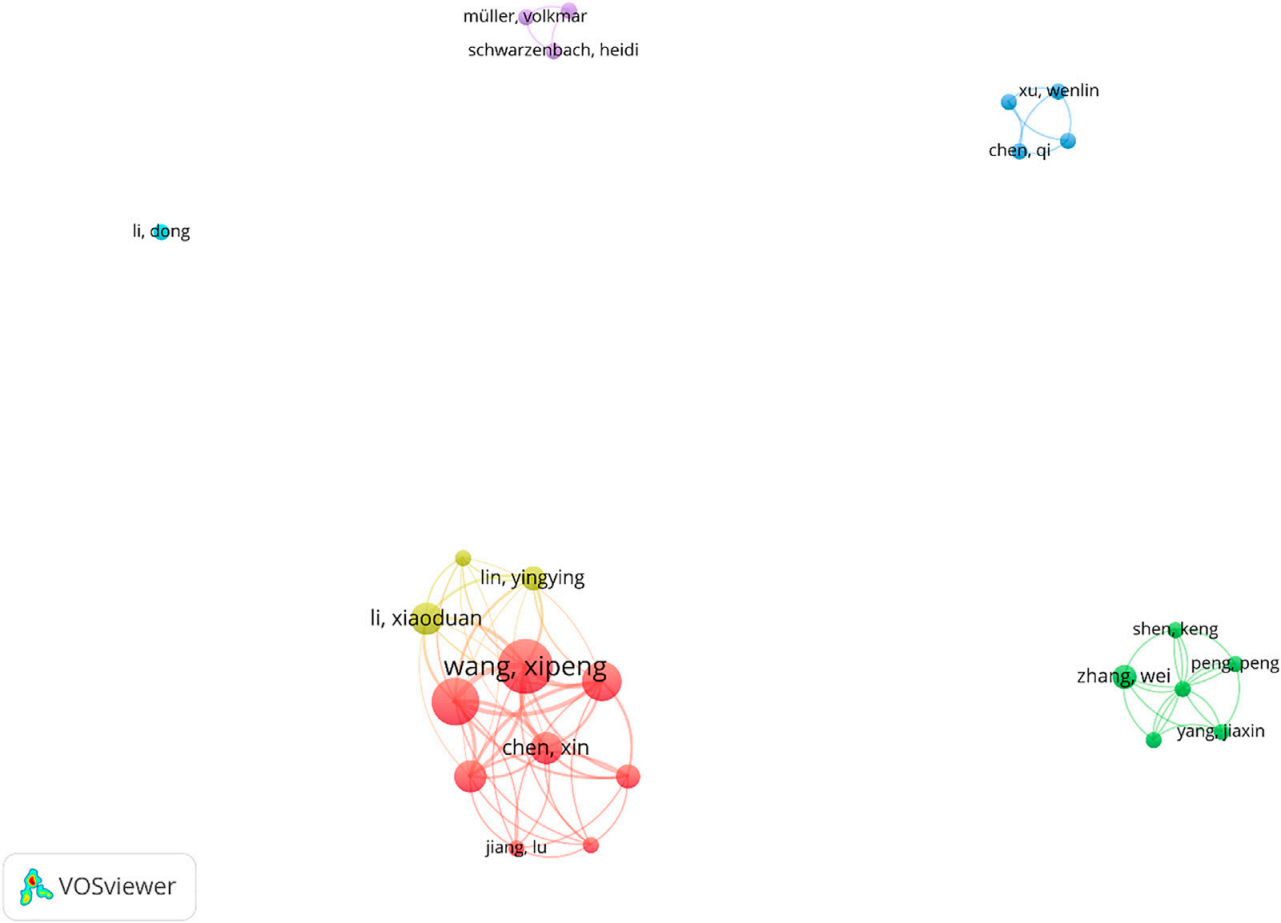
Author co-authorship networks (criteria: authors with at least 2 articles in the field of exosome and OEC).

For authors’ co-authorship analysis, only the authors with at least 2 articles were included in this analysis; so, 25 of 246 authors were entered into the network analysis. As it was demonstrated in Figures 6, 7 groups were resulted by clustering the co-authorships among these 25 authors. Only 2 clusters link together and the rest of them have no link with each other. In this network, a total of 70 links were found between authors.

### 3.5 Citations

A primary analysis of citations showed that the selected articles had been cited 2,101 times, and the mean citation to each article was 53.87 times. 2018 is the year with the maximum number of citations, with 549 citations (26.13%). For articles with the maximum citations, three original articles by Ying et al. (Ying et al., 2016) with 272 citations, Zhou et al. (Zhou et al., 2018) with 230 citations and Meng et al. (Meng et al., 2016b) with 220 citations were first, second, and third, respectively. Table 3 indicates ten articles with the maximum citations in detail. As observed 7 out of these 10 articles focused on the delivery of microRNAs (miRNAs) by exosomes.

**TABLE 3:** The most cited articles in the field of exosome and EOC.

Title	Journal	Year	Citation	References
Epithelial ovarian cancer-secreted exosomal miR-222-3p induces polarization of tumor-associated macrophages	Oncotarget	2016	225	Ying et al. (2016)
Exosomes released from tumor-associated macrophages transfer miRNAs that induce a Treg/Th17 cell imbalance in epithelial ovarian cancer	Cancer immunology research	2018	186	Zhou et al. (2018)
Diagnostic and prognostic relevance of circulating exosomal miR-373, miR-200a, miR-200b and miR-200c in patients with epithelial ovarian cancer	Oncotarget	2016	182	Meng et al. (2016b)
Macrophages derived exosomes deliver miR-223 to epithelial ovarian cancer cells to elicit a chemoresistant phenotype	Journal of experimental and clinical cancer research	2019	181	Zhu et al. (2019)
Exosomes derived from hypoxic epithelial ovarian cancer cells deliver microRNAs to macrophages and elicit a tumor-promoted phenotype	Cancer letters	2018	160	Chen et al. (2018)
Exosomes derived from hypoxic epithelial ovarian cancer deliver microRNA-940 to induce macrophage M2 polarization	Oncology reports	2017	152	Chen et al. (2017)
Exosomal metastasis-associated lung adenocarcinoma transcript 1 promotes angiogenesis and predicts poor prognosis in epithelial ovarian cancer	International journal of biological sciences	2018	99	Qiu et al. (2018)
TGFβ1 in fibroblasts-derived exosomes promotes epithelial-mesenchymal transition of ovarian cancer cells	Oncotarget	2017	85	Li et al. (2017)
Exosomal microRNAs as tumor markers in epithelial ovarian cancer	Molecular oncology	2018	84	Pan et al. (2018)
The emerging roles and therapeutic potential of exosomes in epithelial ovarian cancer	Molecular cancer	2017	76	Li and Wang (2017)

**FIGURE 8:**
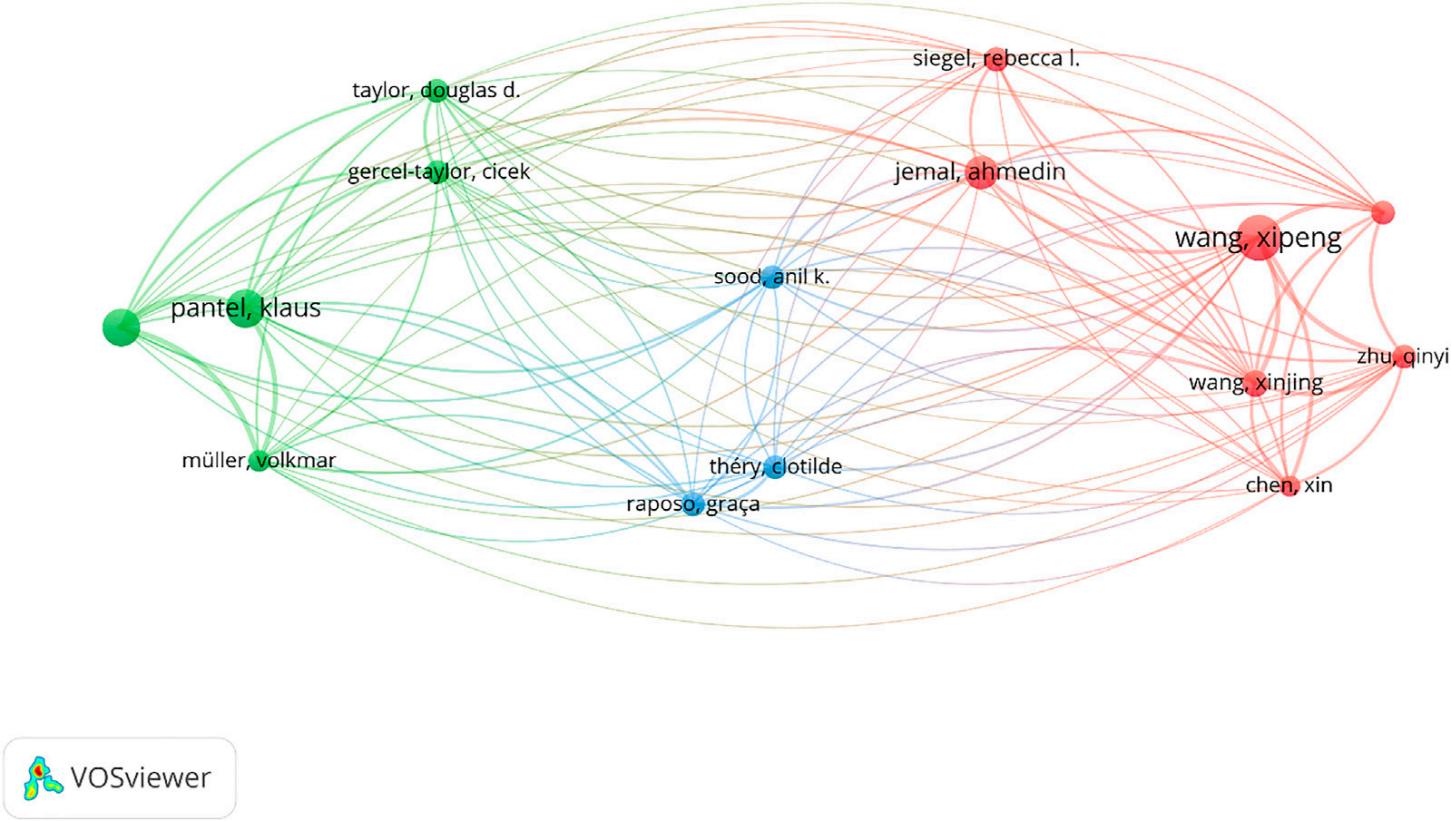
Author co-citation analysis (ACA) (criteria: at least fifteen citations).

On the other hand, the authors’ co-citation analysis (ACA) indicates how often authors of the former articles are co-cited by authors of subsequent articles. These groups cause clusters with central nodes whose size shows the co-citation trend reached by each author. Figure 8 demonstrates a scientific mapping by ACA. The structure contains 10,504 authors, 15 of which meet the threshold recognized in fifteen citations, resulting in 3 clusters. The authors with the maximum citations were: Xipeng Wang (33 co-citations), Klaus Pantel (28 co-citations), and Heidi Schwarzenbach (27 co-citations).

In fact, each cluster in Figure 8 forms a “school of thought” (Zupic and Čater, 2015), which allows us to observe the methods shared between the authors. Cluster 1, which is red, is composed of 7 academics including Xin Chen (15 co-citations), Ahmedin Jemal (24 co-citations), Rebecca l. Siegel (18 co-citations), Xinjing Wang (19 co-citations), Xipeng Wang (33 co-citations), Xiaoli Wu (17 co-citations), and Qinyi Zhu (17 co-citations). This school of thought focuses on different accepts of cancer research (Ying et al., 2016; Siegel et al., 2019). The green cluster 2 is made up of five authors, who focus on cancer biomarkers: Cicek Gercel-Taylor (17 co-citations), Klaus Pantel (28 co-citations), Volkmar Müller (16 co-citations), Heidi Schwarzenbach (27 co-citations), and Douglas D. Taylor (17 co-citations). Among the main lines of this school are tumor-derived exosomes as diagnostic biomarkers of ovarian cancer (Taylor and Gercel-Taylor, 2008) and exosomal microRNAs as EOC markers (Pan et al., 2018). Finally, cluster 3, which is blue, concentrates on 3 researchers: (17 co-citations), Anil K. Sood (17 co-citations), and Clotilde Théry (17 co-citations). These authors address the functions of extracellular vesicles in cancer therapy (Zhao et al., 2022a; Singh et al., 2022).

### 3.6 Journals

**FIGURE 9:**
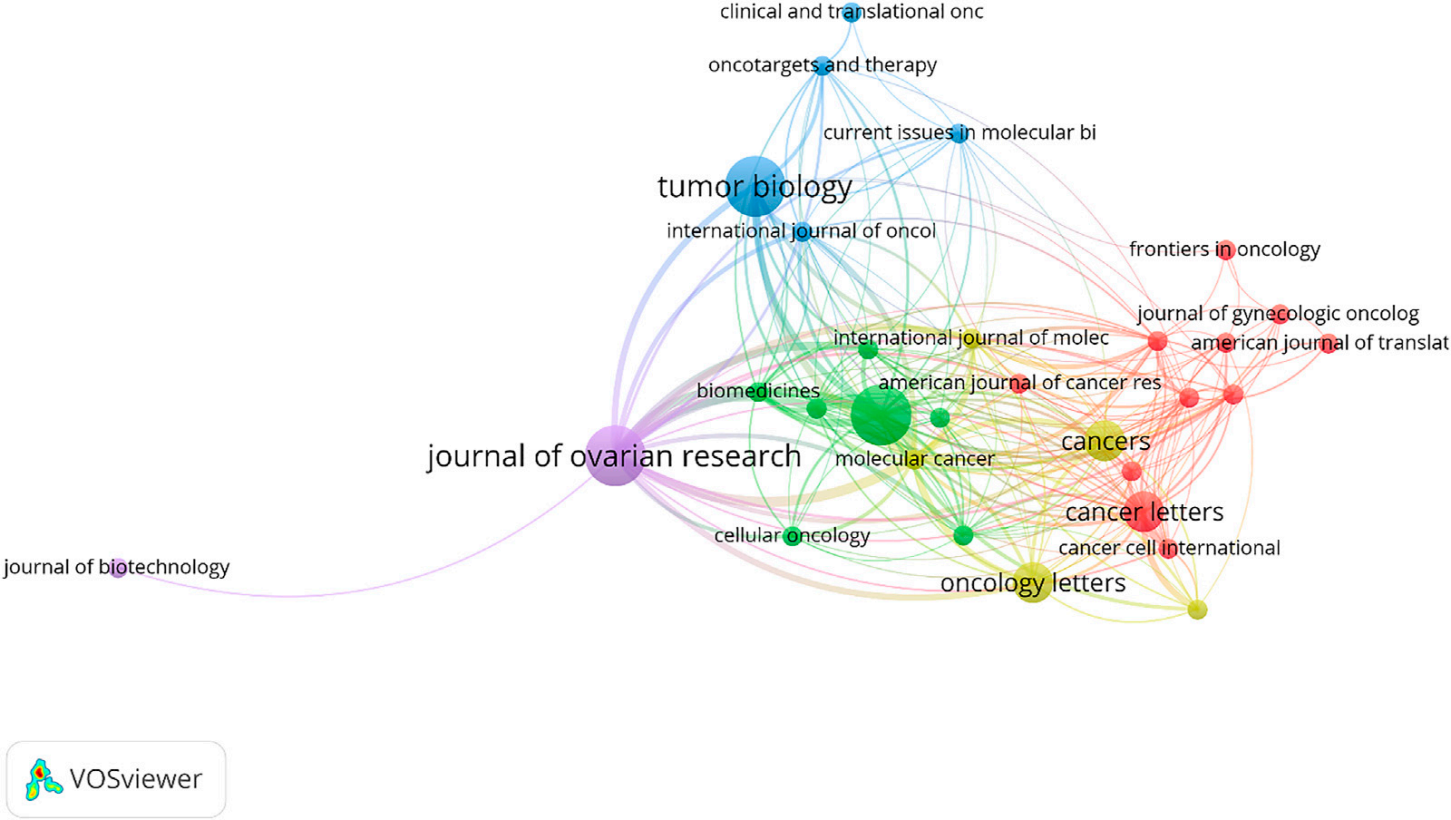
Bibliographic coupling of journals (criteria: journals with at least one article in the field of exosome and OEC).

According to the bibliographic coupling of resources, a total of 30 journals with five clusters were identified (Figure 9). The most productive ones are the Journal of ovarian research, Oncotarget, and Tumor biology, with three articles each. The Oncotarget has the maximum number of citations received by accumulating 492 (Table 4). A total number of 25 journals only published one article in the field of exosome and OEC. 9 out of which (15 out of 39 documents) were published mostly in the United States. 19 out of 25 journals are Q1 suggesting the high quality of the research in this field.

**TABLE 4:** Journals with at least one article in the field of exosome and EOC.

Journal	Papers	Citations	Country	H Index	Q
Journal of ovarian research	3	68	United States	49	1
Oncotarget	3	492	United States	148	1
Tumor biology	3	137	United States	92	1
Cancer letters	2	226	Ireland	192	1
Cancers	2	15	Switzerland	92	2
Oncology letters	2	23	Greec	63	2
American journal of cancer research	1	5	United States	—	—
American journal of translational research	1	14	United States	66	-
Biomedicines	1	2	Switzerland	38	2
Cancer cell international	1	64	UK	62	1
Cancer immunology research	1	186	United States	101	1
Cell death and disease	1	16	United States	128	1
Cellular oncology	1	24	Netherland	47	1
Clinical and translational oncology	1	0	Italy	23	1
Current issues in molecular biology	1	0	Switzerland	54	3
Frontiers in oncology	1	1	Switzerland	102	2
Future oncology	1	0	UK	72	2
International journal of biological sciences	1	99	Australia	98	1
International journal of molecular sciences	1	16	Switzerland	198	1
International journal of oncology	1	64	Greece	128	1
Journal of biotechnology	1	40	Netherlands	164	2
Journal of cell communication and signaling	1	60	Netherlands	47	1
Journal of cellular and molecular medicine	1	7	UK	138	2
Journal of experimental and clinical cancer research	1	181	UK	95	1
Journal of gynecologic oncology	1	29	South Korea	42	1
Molecular cancer	1	76	United States	146	1
Molecular oncology	1	84	Netherlands	97	1
Oncology reports	1	152	Greec	101	1
Oncotargets and therapy	1	19	New Zealand	66	1
Journal of gene medicine	1	1	United States	94	2

### 3.7 Funding sponsors

The main financial sponsors for “exosome and EOC” research was presented in Table 5. The obtained results showed that the National Natural Science Foundation of China was the top funding sponsor with sponsoring 20 projects on this topic.

**TABLE 5:** Sponsors of research in the field of exosome and EOC.

Funder	No of articles
China scholarship council	2
Else kröner-fresenius-stiftung	1
European research council	1
National natural science foundation of China	20
Shanghai municipal commission of health and family planning	2
Ministry of science and technology of the People’s Republic of China	2
Wilhelm sander stiftung	1
Science and technology commission of Shanghai municipality	1
Shanghai hospital development center	1
Shanghai municipal education commission	1
National cancer institute	1
Japan society for the promotion of science	1
Cystic fibrosis foundation	1
Iran National Science Foundation	1
National research foundation of Korea	2
Agencia nacional de investigación y desarrollo	1
National health and medical research council	1

### 3.8 Keywords

**FIGURE 10:**
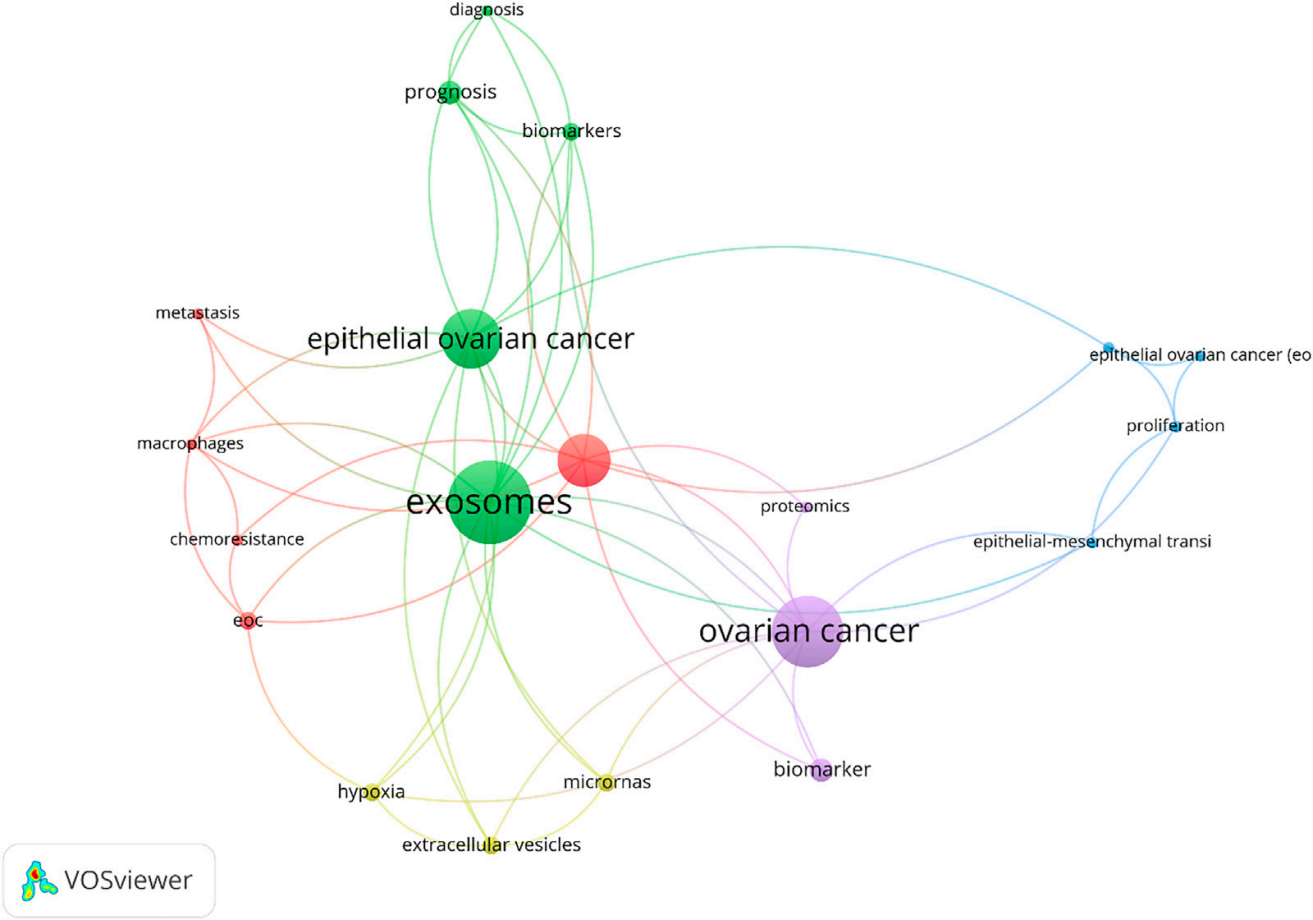
Co-occurrence author keyword (criteria: at least 2 author keyword co-occurrences).

Despite the current relevance of using keywords in some analyses, 3 articles without the keyword section were recognized after reviewing selected articles. In the rest of the articles, the co-occurrence analysis of author keywords is used. For this purpose, at least 2 author keyword co-occurrences were considered as the criteria. 20 out of 114 diverse keywords met the inclusion criteria. As revealed in Figure 10, the analysis of involved keywords resulted in 5 clusters. The keywords with the maximum number of co-occurrences in each group are as follows: Exosomes (14 co-citations), ovarian cancer (12 co-citations), epithelial ovarian cancer (10 co-citations), exosome (9 co-citations), biomarker (4 co-citations), prognosis (4 co-citations), EOC (3 co-citations), biomarkers (3 co-citations), extracellular vesicles (3 co-citations), hypoxia (3 co-citations), microRNAs (3 co-citations), metastasis (2 co-citations), macrophages (2 co-citations), epithelial-mesenchymal transition (2 co-citations), epithelial ovarian cancer (EOC) (2 co-citations), diagnosis (2 co-citations), chemoresistance (2 co-citations), migration (2 co-citations), proliferation (2 co-citations), proteomics (2 co-citations).

## 4 Discussion

The present research mainly purposed to study the international research activities in the field of exosome and EOC from the start of data published in this field up to 15 October 2022. It is emphasized that this research included studies focused on EOC, not ovarian cancer in general and exosomes alone, not microvesicles and extracellular vesicles. There are several bibliometric reviews on different aspects of ovarian cancer (Wang et al., 2016; El Bairi et al., 2021; Gupta et al., 2021; Khedkar et al., 2021; Liu et al., 2022; Xu and Li, 2022) but there is no bibliometric review on EOC and applications of exosomes in EOC. The analysis of productivity by year showed that despite the growth of research interest in exosomes in different fields, research on exosomes and EOC has opened up in recent decades, and it is in the early stages. According to the findings of the current study, China is ranked the first publisher of journal articles in this field. This can be contributed to a large number of highly ranked institutions in publication and more funding sponsors in this region. However, Wang B et al., in 2019 and Wang Y et al., in 2017 stated that the United States ranked as the first producer in the field of exosomes and China ranked second at that time (Wang et al., 2017; Wang et al., 2019). Moreover, Yuanxia Liu et al. reported that the United States and UK are the first and second article producer from inception to 2021 (Liu et al., 2022). When it comes to funding sponsors of the research, Chinese sponsors especially the National Natural Science Foundation of China are impressive. This is since China efforts to impact high-tech technologies and promote their research and development sections and businesses.

Regarding the journal that publishes the articles, the Journal of Ovarian Research is the top publisher of journal articles in the field of exosome and EOC based on our analysis. Wang Y et al. and Wang B et al. stated that Plos One, Journal of Biological Chemistry, and Scientific Reports are the top publishers of articles in the exosome field (Wang et al., 2017; Wang et al., 2019). Furthermore, Bernardo Pereira Cabral et al. reported that Oncotarget led published most cancer-related articles between 2012–2017 (Cabral et al., 2018). All of these journals are among high-ranking journals showing the field of study is novel and high-tech.

Citation analysis showed that 7 out of these 10 most cited articles focused on the delivery of microRNAs (miRNAs) by exosomes. The maximum co-occurrence author keywords showed the research flashpoints in the studied area over the study period. According to this analysis, the academics are interested to work on: 1) Exosomes as prognostic biomarkers of EOC and their role in the proliferation and migration of cells. 2) The role of exosomes in metastasis through different mechanisms; 3) The role of exosomes in epithelial-mesenchymal transition of ovarian cancer cells; 4) The diagnostic role of EVs in EOC; and 5) Conferring chemoresistance in EOC through Exosomal transfer of miRNAs.

## 5 Study limitations and sampling bias

The sampling bias is one of the key limitations of this study. As mentioned, this study focuses on research investigating specifically EOC and exosome. Therefore, sampling criteria excluded the published reports on extracellular vesicles, microvesicles and ovarian cancer, while exosomes are a subclass of extracellular vesicles and microvesicles. Moreover, EOC is one type of ovarian cancer. Therefore, considering exosome and EOC alone cannot make a general conclusion for highly cited or most productive authors in this field of ovarian cancer.

To reduce this limitation, according to citation analysis results which showed most cited articles focused on the delivery of miRNAs by exosomes, an extra search was conducted using the PubMed database on studies in the field of ovarian cancer and exosomal miRNA. 20 other articles other than those found above were found in this field. These articles are related to different countries including the United States, China, Hungary, Australia, Korea, Japan, Germany and the UK. The most cited article in this area is related to the United States with 487 citations, which is considerable. The information related to these articles has been listed in Table 6.

**TABLE 6:** List of articles in the filed of miRNA, exosome and ovarian cancer.

Title	Country	Year	Citation	Ref
Exosomal miRNA confers chemo resistance *via* targeting Cav1/p-gp/M2-type macrophage axis in ovarian cancer	United States	2018	91	Kanlikilicer et al. (2018)
Exosome-Derived microRNA: Efficacy in cancer	United States	2021	5	Padda et al. (2021)
microRNAs as biomarkers of ovarian cancer	China	2020	10	Zhang and Lu (2020)
Expression of CD24 in plasma, exosome and ovarian tissue samples of serous ovarian cancer patients	Hungary	2019	11	Soltész et al. (2019)
Detection of plasma exosomal miRNA-205 as a biomarker for early diagnosis and an adjuvant indicator of ovarian cancer staging	China	2022	7	Zhu et al. (2022b)
Ovarian cancer cell invasiveness is associated with discordant exosomal sequestration of Let-7 miRNA and miR-200	Australia	2014	99	Kobayashi et al. (2014)
miR-139 Controls Viability of Ovarian Cancer Cells Through Apoptosis Induction and Exosome Shedding Inhibition By Targeting ATP7A	China	2019	5	Xiao et al. (2019)
Serum exosomal miRNA-145 and miRNA-200c as promising biomarkers for preoperative diagnosis of ovarian carcinomas	Korea	2019	34	Kim et al. (2019)
Targeted delivery of exosomal miR-484 reprograms tumor vasculature for chemotherapy sensitization	China	2022	7	Zhao et al. (2022b)
MiR-200b is upregulated in plasma-derived exosomes and functions as an oncogene by promoting macrophage M2 polarization in ovarian cancer	China	2021	13	Xiong et al. (2021b)
Quantitative and stoichiometric analysis of the microRNA content of exosomes	United States	2014	478	Chevillet et al. (2014)
Exosomal MicroRNA as Biomarkers for Diagnosing or Monitoring the Progression of Ovarian Clear Cell Carcinoma: A Pilot Study	Japan	2022	3	Horie et al. (2022)
MicroRNA profiling of plasma exosomes from patients with ovarian cancer using high-throughput sequencing	China	2019	8	Zhang et al. (2019b)
Ubiquitous release of exosomal tumor suppressor miR-6126 from ovarian cancer cells	China	2016	31	Kanlikilicer et al. (2016)
Unbiased RNA-Seq-driven identification and validation of reference genes for quantitative RT-PCR analyses of pooled cancer exosomes	United States	2021	8	Dai et al. (2021)
Upregulated expression of serum exosomal miR-375 and miR-1307 enhance the diagnostic power of CA125 for ovarian cancer	China	2019	38	Su et al. (2019)
Exosomes as a potential tool for a specific delivery of functional molecules	Germany	2013	28	Nazarenko et al. (2013)
Exploring the potential of engineered exosomes as delivery systems for tumor-suppressor microRNA replacement therapy in ovarian cancer	United States	2020	37	Kobayashi et al. (2020)
Human CAP cells represent a novel source for functional, miRNA-loaded exosome production	Germany	2019	-	Zeh et al. (2019)
The passenger strand, miR-21-3p, plays a role in mediating cisplatin resistance in ovarian cancer cells	UK	2015	85	Pink et al. (2015)

## 6 Conclusion

The present research findings showed the increasing trend of research activities in the field of exosomes and EOC. The scientific production was made up of 39 journal articles found in the international databases of Scopus, PubMed and Google scholar. China is the main contributor to the publications, institutions, funding, and international collaborations on the studied topic. The United States ranked first in terms of journals published articles on the studied topic. Moreover, results show that the scientists working in this area are mostly interested into apply microRNA delivery using exosomes in EOC contexts. However, it showed be noted that these results only related to studies in the field of EOC, not ovarian cancer in general or nanoscale extracellular vesicles, not all scales extracellular vesicles. These findings could be helpful for academics working in the field of exosomes and EOC to develop their studies and find new pipelines along the way.

## Data Availability

The original contributions presented in the study are included in the article/supplementary material, further inquiries can be directed to the corresponding author.
